# A Cost-Effectiveness and Budget Impact Analysis of AbobotulinumtoxinA in Greece

**DOI:** 10.3390/toxins15090561

**Published:** 2023-09-08

**Authors:** Nikolaos Nomikos, Christos Eleftheriou, Kostas Athanasakis

**Affiliations:** 1 Laboratory for Health Technology Assessment, Department of Public Health Policy, School of Public Health, University of West Attica, 11521 Athens, Greece; 2 Ipsen Mepe, 17456 Athens, Greece

**Keywords:** cost-effectiveness analysis, budget impact analysis, abobotulinumtoxinA, limb spasticity

## Abstract

This study aimed to assess the cost-effectiveness of abobotulinumtoxinA (aboBoNT-A) plus Best Supportive Care (BSC) compared with BSC alone for managing limb spasticity in adult patients in Greece, as well as to conduct a budget impact analysis of the introduction of aboBoNT-A in the Greek healthcare system compared to onabotulinumtoxinA (onaBoNT-A). Clinical studies were utilized to extract data on drug efficacy and patients’ utility, while cost data were collected from Greek sources. The results of the study showed that aboBoNT-A plus BSC was a cost-effective treatment option for both upper and lower limb spasticity in adult patients compared to BSC. Additionally, introducing aboBoNT-A into the Greek healthcare system resulted in cost savings in pharmaceutical spending over a 5-year period. The findings suggest that incorporating aboBoNT-A into the Greek healthcare system could improve patient access to treatment and healthcare resource efficiency, as it is a more economical option compared to onaBoNT-A.

## 1. Plain Language Summary

After a stroke or traumatic brain injury, people often experience muscle and limb stiffness, known as limb spasticity. Treatments for limb spasticity that help to relax stiff muscles include botulinum toxin injections and physiotherapy. Using clinical data from relevant clinical trials in conjunction with population and cost data from Greece, we developed a computer-based model to evaluate the cost and potential benefit of treating adults living with limb spasticity with a specific type of botulinum toxin injection called abobotulinumtoxinA (aboBoNT-A for short) in addition to best supportive care (e.g., physiotherapy) compared with best supportive care alone. We also developed a computer-based model to compare the cost of treating these patients in Greece with two different types of botulinum toxin injection: aboBoNT-A or onabotulinumtoxinA (onaBoNT-A for short). According to our models, combining aboBoNT-A treatment with best supportive care achieved better health outcomes and was more cost-effective than best supportive care alone. This was true for the treatment of adults with upper limb (arm/hand) spasticity and for those with lower limb (leg/foot) spasticity. Our models also found that treatment with aboBoNT-A rather than onaBoNT-A for lower limb spasticity could save the Greek healthcare system over €470,000 in total drug costs over 5 years. For upper limb spasticity, the estimated 5-year saving to the healthcare system was over €114,000. In summary, combining aboBoNT-A treatment with best supportive care could benefit patients, reduce pressures on the Greek healthcare system, and free-up money and time to treat additional patients living with limb spasticity. Further studies would be valuable to confirm these findings and their long-term implications.

## 2. Introduction

Spasticity is a complex neurological condition that arises from an upper motor neuron lesion, which causes a disordered sensory-motor control leading to intermittent or sustained involuntary muscle activation [1]. It is prevalent in many neurological disorders, including stroke, multiple sclerosis, hypoxic brain damage, traumatic brain injury, tumors, and heredodegenerative diseases [2]. The condition often leads to muscle contracture and deformity, potentially reducing function and movement, which may cause disability. Moreover, it has the capacity to affect both pediatric and adult populations. It is frequently categorized according to the distribution of the affected body regions. This classification comprises three main types: focal, regional, and generalized. Focal spasticity pertains to the involvement of a particular body part; regional spasticity refers to the impact on an entire limb, while generalized spasticity denotes the effect on multiple body areas [3].

Spasticity can result in diverse abnormal postures in both the upper and lower limbs, including abnormal arm posture, equinovarus, knee flexion or hyperextension, spastic drop foot with hyper plantar flexion, toe flexion, contracture, pressure ulcers, and subsequent deformities [4,5]. These complications can have a significant impact on the mobility and quality of life of those affected. As a result, spasticity is linked to reduced physical function and diminished ability to carry out daily activities, increased pain, reduced overall health status, and ultimately, a negative effect on the quality of life [6,7]. Additionally, the condition negatively impacts the professional life of patients [8]. Additionally, limb spasticity has a detrimental impact not only on patients but also on their caregivers. Fulfilling caregiving responsibilities often necessitates reduced work hours or even resignation from employment, resulting in financial difficulties [9]. Caregivers frequently experience absenteeism and presenteeism, contributing to the overall caregiving load [10]. This burden encompasses emotional and physical strain faced by caregivers, along with the expenses linked to medical interventions, assistive devices, and home modifications. The associated time and productivity loss related to tending to patients with spasticity can also be substantial [11]. Both patients and their caregivers might encounter out-of-pocket costs, especially for transportation and medical treatments, which further contribute to the economic burden.

The management of the condition involves physical therapy, such as muscle stretching or range of motion exercises, and pharmacotherapy. While several anti-spasticity treatments are available, their lack of specificity and their adverse events limit their usefulness. In this context, local intramuscular injection of botulinum toxin type A (BoNT-A) has emerged as a well-tolerated, first-line treatment option for both upper and lower limb spasticity. BoNT-A injection is supported by a robust body of evidence, and guidelines recommend it as standard clinical practice [5].

AbobotulinumtoxinA (aboBoNT-A) is a type of BoNT-A that is administered through injection. BoNT-A is produced by culturing Clostridium botulinum type A. After several isolation and purification steps, this toxin can be used as medicine and administered to patients through injection. Unlike systemic oral treatments, direct intramuscular administration of BoNT-A through injections reduces the risk of adverse effects caused by organ irritation or absorption and excretion. BoNT-A injections have been found to be effective in enhancing patient mobility and decreasing the likelihood of complications such as joint deformity, tendon shortening, deep vein thrombosis, and pressure ulcers. The evidence shows that aboBoNT-A may produce a more robust response in pediatric patients with upper limb spasticity [12], as well as in adult patients with spasticity in their upper [13] or lower limbs [4], resulting in statistically significant reductions in muscle tone and overall improvement in patient function. BoNT-A offers a sustained alleviation of symptoms, resulting in extended intervals of enhanced mobility, reduced symptom-related anxieties, and heightened self-assurance for patients [11,14,15].

Thus, aboBoNT-A has emerged as an established first-line therapy for upper and lower limb spasticity, providing a safe and effective treatment option for patients. However, in healthcare, it is important to consider not only the clinical effectiveness of an intervention but also its economic impact. Clinical effectiveness refers to the ability of an intervention to achieve its intended health outcomes, while economic impact refers to the costs and benefits of an intervention in relation to its clinical effectiveness.

The economic evidence of an intervention is important because healthcare resources are finite, and decisions need to be made on how to allocate them efficiently. Economic evaluation can help to identify the most cost-effective interventions, which provide the best value for money and can maximize the health benefits for patients. Additionally, it can inform decisions about resource allocation and funding, help to prioritize health interventions and support negotiations with payers and policymakers.

Therefore, it is important to consider both the clinical and economic evidence of an intervention when making decisions about its adoption and implementation. Without economic evidence, it can be difficult to make informed decisions about the use of healthcare resources and ensure that interventions are cost-effective and sustainable in the long run.

Based on the above, the study aimed to assess the cost-effectiveness and budget impact of aboBoNT-A plus BSC compared with placebo plus BSC alone for managing limb spasticity in adult patients in Greece, as well as to conduct a budget impact analysis of the introduction of aboBoNT-A in the Greek healthcare system compared to onabotulnumtoxinA (onaBoNT-A). The objectives of this study include evaluating the economic differences between aboBoNT-A and onaBoNT-A in terms of their impact on pharmaceutical costs, as both regimens can be used for these indications. We use the Greek healthcare system as a field of application of our analysis and take a third-party payer perspective to evaluate aboBoNT-A as an addition to the current treatment of upper and lower limb spasticity in adult patients. Our analysis includes cost-effectiveness and a budget impact approach in both populations.

## 3. Results

The cost-effectiveness analysis for the upper limb spasticity indication found that the mean total treatment cost per patient with aboBoNT-A was €34,020, while supportive therapy alone cost €28,698. The use of aboBoNT-A resulted in an increase in quality-adjusted survival per patient by 0.516 QALYs, with a cost-effectiveness ratio (ICER) of €10,313/QALY gained compared to supportive care (as shown in Table 1).

**Figure 1:**
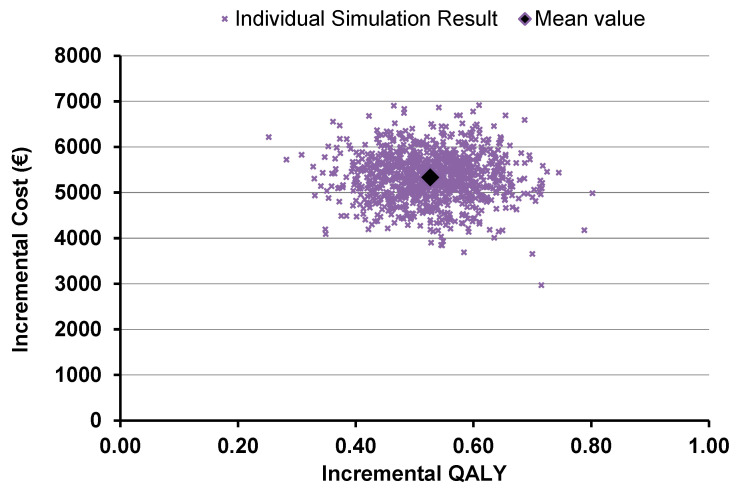
PSA results on upper limb spasticity indication.

**Figure 2:**
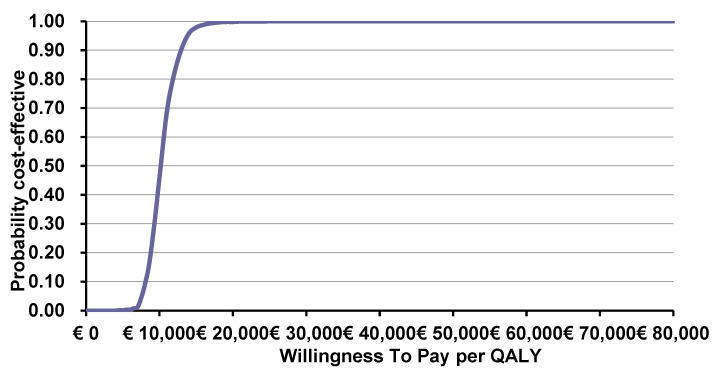
Cost-effectiveness acceptability curve in upper limb spasticity indication.

The probabilistic sensitivity analysis demonstrated that the intervention was cost-effective in all simulations, with a mean ICER of €10,132/QALY gained (as presented in Figure 1). As demonstrated in Figure 2, the intervention is considered 100% cost-effective for a threshold of nearly €20,000 and above.

With regards to adult lower limb spasticity, the analysis demonstrated that the mean total treatment cost per patient with aboBoNT-A was €38,853 versus €29,088 of the supportive therapy, while the QALYs gained from the intervention were 9.66 versus 9.31 of the supportive therapy, resulting in an ICER of €39,465/QALY gained (Table 2).

**Figure 3:**
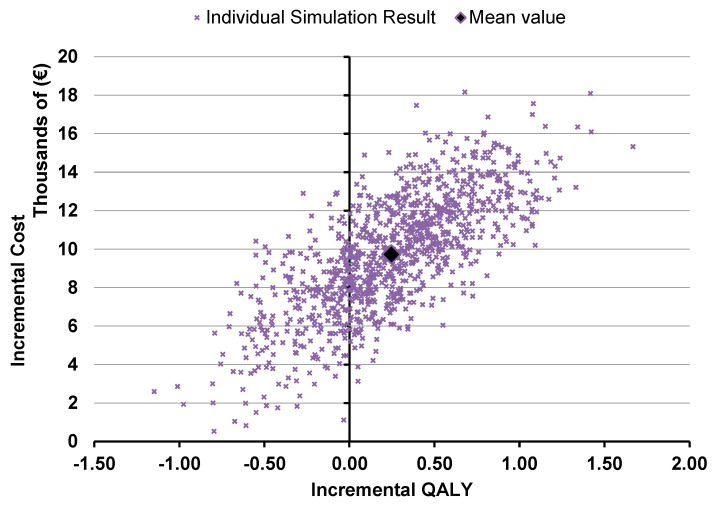
PSA results on lower limb spasticity indication.

**Figure 4:**
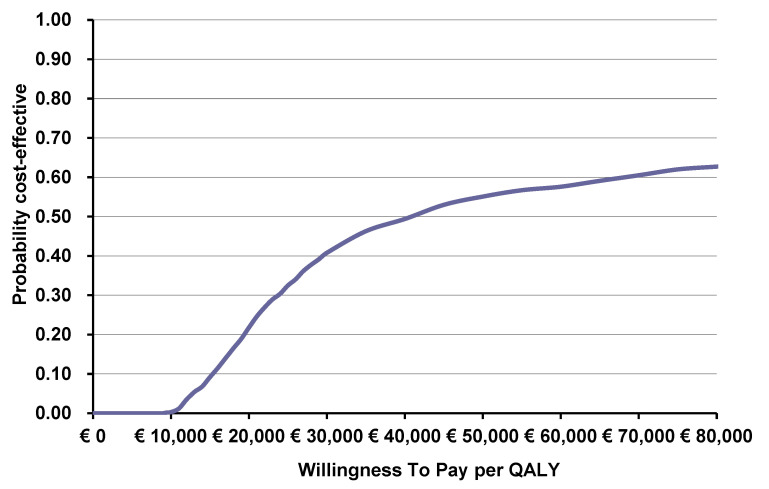
Cost-effectiveness acceptability curve in lower limb spasticity indication.

The probabilistic sensitivity analysis showed that the intervention remained cost-effective in the majority of the simulations, with a mean ICER of €39,442/QALY gained (Figure 3). As shown in Figure 4, the intervention has a 50% chance of being cost-effective for a threshold of €40,000, with the probability of being cost-effective increasing for higher thresholds.

**Figure 5:**
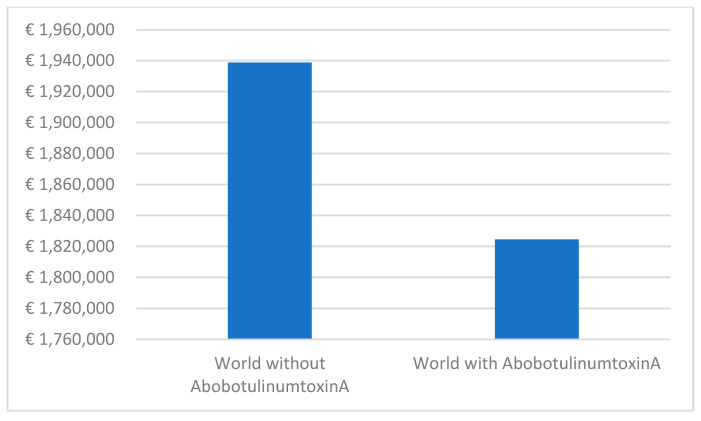
Budget impact analysis results on upper limb spasticity.

The budget impact analysis for upper limb spasticity reveals significant cost savings over a 5-year period. Comparing the two scenarios, the ‘world with aboBoNT-A’ demonstrates an estimated pharmaceutical cost of €1,824,514, while the ‘world without’ presents a higher cost of €1,938,831 (Figure 5). This difference amounts to a total savings of 5.9% in pharmaceutical expenses for upper limb spasticity. Furthermore, the monthly cost per patient experiences a downward trend, decreasing from €122.4 in the initial year to €108.0 in the fifth year.

**Figure 6:**
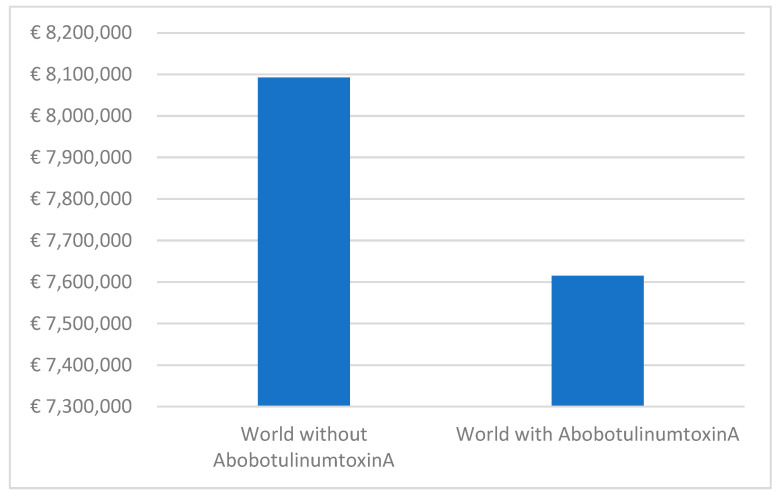
Budget impact analysis results on lower limb spasticity.

In the context of lower limb spasticity, the scenario ‘world without’ translates to pharmaceutical costs of €8,072,295, whereas the scenario ‘world with’ reflects reduced costs of €7,615,141 (Figure 6). This demonstrates a tangible reduction in expenses over the 5-year period, with the highest savings realized in the fifth year when the utilization of aboBoNT-A therapy is at its peak for lower limb spasticity. The average monthly cost per patient consistently decreases, highlighting the cumulative financial benefits associated with aboBoNT-A therapy for both upper and lower limb spasticity.

## 4. Discussion

Based on the results of the CEA for the upper limb spasticity indication, it can be concluded that the use of aboBoNT-A is a cost-effective treatment option compared to supportive care alone. The ICER of €10,313/QALY gained shows that the additional cost of the treatment per QALY gained is reasonable. The probabilistic sensitivity analysis confirms that the intervention is cost-effective in all simulations, with a mean ICER of €10,132/QALY gained. This indicates that the use of aboBoNT-A is a favorable and efficient option for patients with upper limb spasticity. Similarly, the analysis for the lower limb spasticity indication also showed that aboBoNT-A is a cost-effective treatment option compared to best supportive care alone. The ICER of €39,465/QALY gained is higher than that of upper limb spasticity but still falls within the acceptable range. The probabilistic sensitivity analysis also confirmed the cost-effectiveness of the intervention in the majority of simulations, with a mean ICER of €39,442/QALY gained. This suggests that the use of aboBoNT-A is a favorable and efficient option for patients with lower limb spasticity.

The present study found aboBoNT-A to be a cost-effective option for the treatment of lower and upper limb spasticity in adults in Greece. Our study is in line with other studies in different countries, which have also reported that aboBoNT-A is a cost-effective treatment option for the treatment of lower and upper limb spasticity. For example, in Australia, aboBoNT-A was found to be a cost-effective treatment for lower limb spasticity in adults [16]. Additionally, in the United Kingdom, aboBoNT-A was reported to dominate placebo plus BSC as a treatment option for upper limb spasticity in adults [17] and was identified as a cost-effective approach for the management of wrist and hand disability resulting from upper-limb post-stroke spasticity in Scotland [18]. Furthermore, studies conducted in the UK have also found that aboBoNT-A was a cost-effective strategy compared to placebo plus BSC for the treatment of post-stroke spasticity [19] and was identified as the dominant strategy compared to onaBoNT-A for all indications [20].

Our study examined the financial impact of aboBoNT-A treatment for upper and lower limb spasticity and found that it resulted in cost savings in pharmaceutical spending over a 5-year period. These findings are consistent with other studies, such as one conducted in the UK where aboBoNT-A was found to be less expensive per patient per year than best supportive care for upper limb spasticity, while onaBoNT-A and incobotulinumtoxinA were more expensive [21]. However, a study in Germany showed that the use of aboBoNT-A for upper limb spasticity in adults increased costs compared to usual care but was still less expensive than its comparator. Another study in Germany suggested that increasing the use of aboBoNT-A compared to onaBoNT-A and incobotulinumtoxinA for upper limb spasticity could potentially reduce the total cost of treatment [22].

Thus, using aboBoNT-A could lead to cost savings and potentially increase patient access to treatment while improving healthcare resource efficiency. For example, with a hypothetical annual pharmaceutical budget of €300,000 in Greece, aboBoNT-A could provide treatment for an additional 85 adult patients with upper limb spasticity each year. Similarly, with a hypothetical annual pharmaceutical budget of €1,000,000, the introduction of aboBoNT-A in the Greek market could enable 284 more patients with lower limb spasticity to receive treatment annually.

## 5. Conclusions

In conclusion, the findings of this study suggest that aboBoNT-A can be a cost-effective and efficient treatment option for patients with upper and lower limb spasticity after stroke or brain trauma. The results of the sensitivity analysis demonstrated the robustness of the findings and the potential for the drug to remain cost-effective in various scenarios. The BIA also revealed the potential for significant savings for the Greek healthcare system and increased access to treatment for patients, as under the same budget, more patients can be treated with aboBoNT-A. These findings have important implications for healthcare policy and decision-making in Greece and could help improve the quality of life of patients with ULS and LLS. Further research may be needed to confirm these findings and explore the long-term effects of aboBoNT-A treatment.

## 6. Materials and Methods

### 6.1. Models Structure

A cost-effectiveness analysis (CEA) was conducted to assess the cost-effectiveness of aboBoNT-A plus BSC compared with BSC alone. Concurrently, a Budget Impact Analysis (BIA) was performed to gauge the monetary implications of integrating aboBoNT-A into the healthcare landscape of Greece, drawing a comparison with the financial effects of onaBoNT-A. For our analysis, we use and adapt to the Greek healthcare setting four pharmacoeconomic models conducted in Excel 2016 (Microsoft Corporation, Redmond, WA, USA, 2016). We use a CEA model and a BIA across two indications/populations: adults with lower limb spasticity and adults with upper limb spasticity.

In principle, each cost-effectiveness model aims to assess the clinical and economic implications (costs and health benefits) of adding aboBoNT-A BSC compared to placebo and BSC for patients with spasticity. Furthermore, both CEA models are tailored to align with the characteristics of populations as observed in the clinical trials. These models are further enhanced through the integration of pertinent population and cost data specific to Greece. Conversely, the BIA models aimed to estimate the impact on third-party payers resulting from the introduction of aboBoNT-A in Greece. Detailed information regarding the models for each of the two populations is provided below.

### 6.2. Cost-Effectiveness Models

#### 6.2.1. Upper Limb Spasticity in Adults: Modelling Approach

The model is designed for the population of the NCT01313299 [23] clinical trial. This trial included specific patient inclusion criteria, comprising ambulatory adults who had undergone a stroke or brain injury and had a Modified Ashworth Scale score of ≥2. On the contrary, the exclusion criteria encompassed patients with a history of botulinum toxin treatment of any type within 4 months before study entry for any condition, those who had initiated physiotherapy less than 4 weeks before inclusion, individuals with a history of previous surgery, alcohol use, phenol treatment in the upper limb, and neurological or neuromuscular disorders that could potentially interfere with the protocol evaluations. These criteria were pivotal in ensuring the relevance and applicability of our study’s findings within the context of the NCT01313299 [23] trial population.

The model for adult upper limb spasticity (AUL) employs health states based on spasticity severity as determined by the Disability Assessment Scale (DAS)—a validated instrument for assessing changes in upper limb functionality. The DAS consists of four levels, ranging from 0 (no disability) to 3 (severe disability). The model begins with a population of patients that match the baseline characteristics of patients in the aboBoNT-A upper limb spasticity clinical trial program in terms of age, sex, and DAS level distribution. Transition probabilities are derived from changes in DAS level experienced by patients in the trial and are applied during 12-week cycles in line with the aboBoNT-A product label.

During each cycle, treatment and other healthcare costs, as well as utility values specific to the DAS level, are applied. Costs and quality-adjusted life years (QALYs) are accumulated over the specified time horizon. Treatment discontinuation is modeled uniformly across all health states after year 1, with patients remaining in the health state achieved at the time of discontinuation until death. Results are expressed in terms of the incremental cost, incremental QALYs, and the incremental cost-effectiveness ratio. The time horizon of the model was set at a lifetime with a maximum of 99 years. A discount rate of 3.5% is applied to costs and outcomes, following the NHS NICE guidelines [24], as there are no relevant Greek official guidelines. The costs under consideration pertain to the year 2022.

#### 6.2.2. Lower Limb Spasticity in Adults: Modelling Approach

The model is tailored to the population of the NCT01249404 [14] clinical trial, with inclusion criteria encompassing ambulatory adult patients aged 18 to 80 years old who have experienced a stroke or brain injury, exhibiting muscle tone intensity greater than or equal to 2, as measured on the Modified Ashworth Scale. In contrast, exclusion criteria involved individuals with fixed contractures, those who had initiated physiotherapy within 4 weeks before entry, patients with a history of previous surgery or treatment involving phenol and/or alcohol in the lower limb, and those with neurological or neuromuscular disorders that could potentially impede the protocol evaluations. These criteria were crucial in ensuring the applicability and relevance of our study’s findings within the context of the NCT01249404 [14] trial population.

The model for adult lower limb spasticity (ALL) uses health states determined by outcome measures assessed by the Physician’s Global Assessment (PGA), which incorporates health states based on spasticity severity and improvement. The PGA is a validated tool for evaluating an individual’s response to treatment following the injection of the study toxin, and we collapsed its nine levels into binary categories of “responder” and “non-responder” for the CEA model. We selected the PGA as the measure to assess outcomes in the economic model based on statistical analyses that demonstrated its reliable ability to map to health utility measures.

The model begins by selecting a population of patients whose baseline characteristics match those of the patients enrolled in the aboBoNT-A lower limb spasticity clinical trial program in terms of age and sex. To simulate treatment outcomes, transition probabilities are applied during 12-week cycles based on changes in the level of outcome measure experienced by patients in the trial, in line with the aboBoNT-A product label. During each cycle, treatment and other healthcare costs, as well as utility values (based on the PGA), are considered.

To model treatment discontinuation, patients are assumed to discontinue uniformly across all health states after year 1. Patients are then assumed to remain in the health state achieved at the time of discontinuation until death. Results are expressed in terms of the incremental cost, incremental QALYs, and the incremental cost-effectiveness ratio. The time horizon of the model was set at a lifetime with a maximum of 99 years. A discount rate of 3.5% is applied to costs and outcomes, following the NICE guidelines, as there are no relevant Greek official guidelines [24]. The costs being evaluated refer to 2022.

### 6.3. Efficacy and Transition Probabilities

#### 6.3.1. Upper Limb Spasticity Model

In the economic evaluation of adult upper limb spasticity treatments, a Markov model was utilized to capture the dynamic nature of patient health states. The improvement in disability based on the DAS health states was considered as the treatment efficacy. Patients who experienced reduced disability were considered to have improved, whereas those who remained in the previous health state or experienced increased disability were considered to have worsened. The efficacy of aboBoNT-A treatment was derived from the NCT01313299 and NCT01313312 clinical trial data, using the randomized trial data from baseline through 12 weeks and the open-label extension data through 48 weeks. We assumed that 10% of patients in each health state discontinued treatment after the first year. BSC treatment efficacy was derived from the NCT01313299 [23] Trial for outcomes from baseline to 12 weeks, and beyond 12 weeks, patients were assumed to remain in the health state they were in at the end of the 12-week clinical trial period.

The transition probabilities for each health state were initially derived from patient-level data collected during the NCT01313299 [23] randomized trial. These data were used to analyze how patients move through various health states in the model. Each patient starts with a baseline probability of being in one of the health states, and the initial health state distribution was assigned using baseline data from the clinical trial. Transition probabilities for each dose of aboBoNT-A were derived from the NCT01313312 [25] Trial at 12 weeks, and data for transition probabilities beyond 12 weeks were obtained from the NCT01313299 [23] study. The model assumes that patients remain in the health state they were in at the end of the trial for the lifetime horizon, as no study data about health state progression beyond the final trial endpoint are available.

#### 6.3.2. Lower Limb Spasticity Model

In the evaluation of aboBoNT-A treatment efficacy for adult lower limb spasticity, a Markov model was employed to analyze patient transitions between different health states as defined by the PGA. Specifically, patients who experienced a reduction in disability, such as moving from moderate to mild disability, were classified as improved, while those who remained in the same health state or experienced an increase in disability were considered to have worsened. To determine the treatment efficacy, data from the NCT01249404 [14] and NCT01251367 [14] clinical trials were used. The randomized trial data was used from baseline to 12 weeks, and data from the NCT01251367 trial was used for up to 48 weeks. In the base case, treatment efficacy was extrapolated from the observed data for the lifetime of the patient, assuming that the transitions between weeks 36 and 48 were repeated for the remaining time horizon. Additionally, it was assumed that 10% of patients in each health state discontinued treatment after the first year. The efficacy of placebo plus BSC was derived from the placebo arm of the NCT01249404 [14] trial and was evaluated based on outcomes from baseline to 12 weeks. Beyond 12 weeks, it was assumed that patients remained in the same health state they were in at the end of the 12-week clinical trial period.

Transition probabilities were calculated based on patient-level data collected during the ALL NCT01249404 [14] randomized trial to determine how patients move through different health states in the model. The PGA-based model did not consider an initial health state distribution because it measures the improvement or worsening of spasticity symptoms from the baseline visit. Transition probabilities for each aboBoNT-A dose were derived from the NCT01249404 [14] Trial at 12 weeks. Data for transition probabilities beyond 12 weeks were obtained from the NCT01251367 [14] Trial (for weeks 24–48). Patients were assumed to remain in the health state they were in at the end of the trial for the lifetime horizon since there was no study data on health state progression beyond the final trial endpoint.

### 6.4. Resource Use and Costs

To complement our cost data, we collected information on resource utilization in Greece through a literature search. Our model incorporated diverse cost parameters, such as drug procurement and administration, medical exams and procedures, medical visits, and physiotherapy. We sourced the cost data for these parameters from Greek references. Specifically, we obtained the cost of examinations from the EOPYY reimbursement list [26], the cost of hospitalizations from the Greek DRG system [27], which we adjusted for hospital staff salaries [28], and the cost of drugs from the Greek Ministry of Health [29]. Table 3 presents all the cost parameters included in the CEA models.

### 6.5. Quality of Life

Utility weights for treatment responders and non-responders, which were obtained from clinical trials after converting SF-36/SF-6D health domains into EuroQol 5D (EQ-5D) instruments, are presented in Table 4 and Table 5 for AUL and ALL, respectively.

Additional parameters regarding the cost-effectiveness models can be found in Appendix A (Table A1 and Table A2 for adult upper limb spasticity and upper limb spasticity cost-effectiveness model, respectively).

### 6.6. Uncertainty

To determine the reliability of the model results in the face of varying assumptions, a Monte Carlo simulation is employed to conduct a probabilistic sensitivity analysis (PSA). This entails simultaneously altering all parameters in the model using randomly selected estimates from their respective distributions. The simulation is repeated 1000 times, and the ICER values are documented for each iteration. Additionally, the simulation-level results are depicted in visual form through a cost-effectiveness plane and a cost-effectiveness acceptability curve.

### 6.7. Budget Impact Model

For the BIA, we use two Budget impact models, which were developed to determine the yearly cost per patient and overall impact (cumulative costs) of introducing aboBoNT-A in Greece. The analysis was conducted from the viewpoint of the third-party payer, namely the Greek National Organization for Health Care Services Provision (EOPYY). The analysis spanned up to 5 years, and no discounting was employed in the results, consistent with the International Society for Pharmacoeconomics and Outcomes Research (ISPOR) guidelines for conducting budget impact analyses [30].

The budget impact model assesses direct medical costs under two scenarios: with and without aboBoNT-A. The model predicts a gradual increase in aboBoNT-A in market share over a 5-year time horizon for both indications against its comparator, onaBoNT-A, starting from zero market share in the first year (Table 6).

The cost data includes only the drug cost of BoNT-A therapy, specifically aboBoNT-A and onaBoNT-A, as well as the administration cost. It was assumed that the utilization of other healthcare resources would not change with the shift in therapy. The cost of BoNT-A therapies was extracted from the Greek Ministry of Health [29].

## Figures and Tables

**Table 1:** Analysis results on upper limb spasticity.

Incremental Results	Pooled Dose
Total Direct Healthcare Costs	€5322
HRQoL (QALY)	0.5160
ICER (cost per QALY)	€10,313

**Table 2:** Analysis results on lower limb spasticity.

Difference (Lifetime Time Horizon)	Pooled Dose
Total Direct Healthcare Costs	€9764.51
HRQoL (QALY)	0.2474
ICER (cost per QALY)	€39,465.39

**Table 3:** Cost parameters in the CEA models, in Euros (€).

General Practitioner or Specialist Visit Cost	10.00
Laboratory Test Cost	14.07
Physiotherapy Cost	15.00
Centrally acting Medication Cost	3.00
Peripherally acting Medication Cost	1.61
Anticonvulsant Medication Cost	5.09
Anxiolytic Medication Cost	1.86
Hypnotic Medication Cost	2.19
Analgesic Medication Cost	3.35
Non-aspirin NSAID Medication Cost	2.38
Antidepressant Medication Cost	5.76
Hospitalization Cost per Day	489.50
Cost of abobotulinumtoxinA per vial	155.7

**Table 4:** Quality of life parameters in EQ-5D in the adult upper limb spasticity cost-effectiveness model.

Quality of Life (Utility)	EQ-5D	Source
No disability	0.78	NCT01313299
Mild disability	0.73	
Moderate disability	0.67	
Severe disability	0.61	

**Table 5:** Quality of life parameters in EQ-5D in the adult lower limb spasticity cost-effectiveness model.

Quality of Life (Utility)	EQ-5D	Source
Non-Responder	0.4918	NCT01249404
Responder	0.54	

**Table 6:** Market share of AbobotulinumtoxinA.

Year	Market Share in ALL	Market Share in AUL
1	0%	0%
2	10%	10%
3	20%	20%
4	30%	30%
5	40%	40%

## Data Availability

Restrictions apply to the availability of these data since the data underlying this publication were provided by Laboratory for Health Technology Assessment, Department of Public Health Policy, School of Public Health, University of West Attica, 11521 Athens, Greece, under contract to Ipsen.

## Appendix A

**Table A1:** Additional paraments of the cost-effectiveness analysis for upper limb spasticity.

**Model Operating Parameters**	
Cycle length (weeks)	12.00
Time Horizon (years)	47.00
Costs discount rate	3.50%
Outcomes discount rate	3.50%
**Demographics**	
Age (years)	52.93
Female (%)	35.80%
**Initial Health State Distributions**	
No disability	0.00%
Mild disability	0.00%
Moderate disability	43.80%
Severe disability	56.20%

**Table A2:** Additional paraments of the cost-effectiveness analysis for lower limb spasticity.

**Model Operating Parameters**	
Cycle length (weeks)	12.00
Time Horizon (years)	47.00
Costs discount rate	3.50%
Outcomes discount rate	3.50%
**Demographics**	
Age (years)	53
Female (%)	43%
